# Early maladaptive schemas as mediators of pronatalist pressures: comparative study of psychological distress in child-free women and mothers

**DOI:** 10.1192/bji.2025.10093

**Published:** 2026-05

**Authors:** Maria Evangelopoulou, Stavroula I. Bargiota, Ioanna Ierodiakonou-Benou, Myrto Samara

**Affiliations:** 1 3rd Department of Psychiatry, Faculty of Medicine, School of Health Sciences, Aristotle University of Thessaloniki, Greece; 2 Department of Psychiatry, Faculty of Medicine, School of Health Sciences, University of Thessaly, Greece

**Keywords:** Early maladaptive schemas, pronatalist pressures, psychological distress, motherhood, childlessness

## Abstract

**Background:**

Societal pronatalist ideologies link womanhood to motherhood and create social pressures that often cause psychological distress when unmet. While previous research has documented the impact of these external pressures, much less is known about how women’s internal belief systems shape their responses to societal expectations. Recognising this gap, we examined the perspectives and concerns about motherhood and childlessness among child-free Greek women, their psychological distress and the potential mediating role of early maladaptive schemas (EMS) in this relationship. To provide context, we compared these findings with those of mothers, considering both their mental health and their assessments of similar issues after becoming parents.

**Method:**

We conducted an online, cross-sectional survey comparing child-free Greek women and mothers aged 30–50 years. Through custom-designed questionnaires, we evaluated concerns about motherhood and childlessness among child-free women, and we asked the mother group to assess the same aspects post-motherhood. Mental health was assessed using Depression, Anxiety and Stress Scale 21, while EMS were measured with the Young Schema Questionnaire. We used mediation analysis to examine whether EMS mediated the connection between pronatalist pressures and mental health outcomes.

**Results:**

The study included 1341 women, comprising 503 without children and 838 with children. Child-free women, who were predominantly involuntarily child-free, tended to be younger, more educated and had lower household incomes than mothers. They also reported more diverse sexual orientations, higher levels of depression and anxiety and elevated scores on EMS, which appear to exacerbate distress caused by societal pronatalist pressures. In contrast, mothers were typically older and more financially stable. Although they reported fulfilment from motherhood, they also faced challenges, including demanding caregiving responsibilities and financial or logistical barriers to having more children. Mothers’ psychological profiles were characterised by lower EMS scores and less overall distress.

**Conclusions:**

This study revealed distinct demographic and psychological profile differences between child-free women and mothers. Our findings underscore that women’s mental health in the context of reproduction is shaped by a complex interaction of social pressures, psychological schemas and structural factors. Specifically, EMS – particularly in the domains of Emotional Deprivation, Mistrust/Abuse and Vulnerability to Harm – were identified as a key mechanism, mediating the relationship between pronatalist pressures and psychological distress. Furthermore, occupational disparities emerged as a significant stressor, affecting both child-free women in high-pressure careers and mothers who were home-makers.

Motherhood has long been closely connected to female identity, influenced by biological, social and cultural factors.^1^ In many societies, including Greece, it is often seen as an inherent and expected role for women, based on their biological ability to bear and nurture children.^2^ Women additionally face pressure from the ‘biological clock’ narrative, which reinforces the idea that motherhood is a ‘timed’ obligation, while childlessness is considered a failure or loss rather than a valid life choice.^3,4^ Those without children often encounter stigma, social isolation and feelings of guilt,^5^ while the uncertainty surrounding reproductive choices can also adversely affect overall well-being.^6,7^ Conversely, mothers frequently experience stress from the expectations of ‘intensive mothering’^8,9^ and the demand for self-sacrifice, even while deriving a sense of fulfilment.^10^ These dynamics highlight the mental health implications of societal pressures on motherhood; however, there is still a need to better understand the cognitive processes involved that govern how these pressures are processed and experienced, such as the role of early maladaptive schemas (EMS).

EMS are broad, pervasive patterns of thinking, feeling and behaving that develop during childhood or adolescence due to unmet core emotional needs (e.g. safety, acceptance, autonomy). These schemas act as stable frameworks, persist into adulthood and shape how individuals interpret themselves, others and the world.^11,12^ EMS may influence how a person perceives and copes with stressors, such as societal expectations, leading often to increased distress.^13^ In that sense, EMS mediate the relationship between external societal pressures and mental health outcomes because they shape the internal processing of these external demands. For example, a woman with an EMS related to defectiveness may perceive societal pressure to become a mother as personal rejection or failure, leading to depression or anxiety. Thus, EMS provide a key psychological link between early life experiences and adult mental health responses to current social stressors.

Recognising EMS creates opportunities for targeted interventions. Because mental health issues may stem from external pressures and problematic internal thoughts, methods such as schemas and cognitive–behavioural therapy can address these core schemas. By transforming negative beliefs (such as emotional deprivation, mistrust and defectiveness), clinicians can help women develop healthier patterns, which reduce distress related to reproductive choices and societal expectations. This highlights the importance of interventions beyond basic stress management, emphasising cognitive restructuring and emotional processing to help women independently navigate their reproductive identity.

This study examined EMS as mediators of pronatalist pressures among Greek women aged 30–50 years, including both child-free women and mothers. We chose the 30- to 50-year age range because this period is critical for reproductive decisions, marked by societal and biological pressures related to motherhood. Women in their 30s and 40s often face concerns about fertility decline and the biological clock narrative, which influence their attitudes to, and stress surrounding, parenthood. This age group also balances reproductive choices with personal, relational and professional demands, making it highly relevant for studying the psychological impact of pronatalist pressures.

This study advances understanding by contrasting women’s assessments of motherhood before and after becoming parents, and by examining how EMS modulate pronatalist pressures. To our knowledge, it represents the first systematic investigation to frame EMS as dynamic mediators in this context, bridging cognitive frameworks with sociocultural influences. By illuminating how these schemas shape women’s navigation of reproductive expectations, the findings offer novel pathways for interventions that empower women to make autonomous choices in pronatalist societies, prioritising psychological and systemic support aligned with their evolving realities.

## Method

This cross-sectional study used a dual-group design to explore psychological distress, societal pressures and EMS among Greek women aged 30–50 years, including child-free women and mothers. We use the term ‘child-free’ throughout this study as a neutral descriptor to avoid the negative connotations of ‘childless’. However, this should not be interpreted as representing a homogeneous group of intentionally child-free women. In our study, we combined women who were both voluntarily and involuntarily child-free into a single group of women without children; they were grouped together because of the complex and overlapping nature of their experiences. Many women fluctuate between wanting children and choosing to remain child-free due to various life circumstances. This approach enables the study of shared psychological mechanisms, such as EMS, that influence responses to societal pressures regardless of reproductive intentions. The group of women with children included biological, adoptive and foster parents.

Data were collected via online, self-administered questionnaires written in the Greek language, from March to June 2022, with participants recruited nationally through social media, parenting forums and women’s health organisations to ensure diverse geographical and socioeconomic representation.

All participants completed anonymous online questionnaires to gather detailed information. Partial responses were omitted. A demographic questionnaire collected information on age, number of siblings, residence, sexual orientation, marital status, education level, religion, profession, income, gynaecological history and COVID-19 vaccination status. The reason for collecting COVID-19 vaccination status was to explore fertility concerns linked to the vaccine, a common public worry during the pandemic. Knowing participants’ vaccination status helped examine how these fears might affect reproductive decisions and mental health among women of child-bearing age. Some participants cited fertility fears as a vaccination barrier, reflecting broader anxieties about motherhood and child-bearing. Ethnicity was not included in the demographic data due to the relative ethnic homogeneity of the Greek population in our sample, making meaningful analysis by ethnicity impractical and less relevant in the specific sociocultural context of Greece.

Participants subsequently completed a custom scale (presented in the Supplementary material available at https://doi.org/10.1192/bji.768.2025.10093). The custom scale was primarily developed based on extensive clinical practice and the demands encountered in therapeutic settings with women facing reproductive concerns. This was complemented by an extensive literature review and preliminary qualitative feedback from women reflecting on their experiences, ensuring that the items captured relevant cognitive and emotional aspects of motherhood and childlessness in real-world clinical contexts. Child-free women were asked to reflect on their thoughts and feelings about motherhood, evaluating both the possibility of having a child and remaining child-free. Using a five-point Likert scale,^14^ they rated the importance of partner compatibility, medical procedures, biological clock anxiety, anticipated life changes and perceived responsibility burdens. The scale also evaluated concerns associated with not having children, including feelings of unfulfilment, external expectations, a sense of missing out and fear of future regret. These specific childlessness concerns were chosen because they represent core psychological and social challenges consistently reported in the literature on reproductive health and infertility. Research indicates that women experiencing childlessness often face grief, social stigma, identity disruption, anxiety and depression linked to worries about finding a partner, the pressure of declining fertility with age and fears about future regret.^15,16^ These concerns are well-documented stressors in both involuntary and voluntary childlessness, influencing both mental health and quality of life.

Mothers completed a custom scale to evaluate their desire for another child and perceptions of motherhood (see Supplementary material). The goal was to understand how they assessed partner suitability, life changes, responsibilities, feelings of achievement, social expectations, completion and regret after motherhood.

Standardised psychological tools included Young Schema Questionnaire Short-Form 3 (YSQ-S3) on a 6-point Likert scale. YSQ-S3 was used to assess 18 EMS (emotional deprivation, abandonment, mistrust/abuse, social isolation, defectiveness, failure, dependence, vulnerability to harm, enmeshment, subjugation, self-sacrifice, emotional inhibition, ‘unrelenting standards’, entitlement, insufficient self-control, approval research, negativity, punitiveness).^17^ Depression Anxiety Stress Scale 21 (DASS-21) was used to assess psychological distress with 21 items, 7 for each subscale, rated on a 4-point severity scale.^18^ According to established cut-offs, the normal range for depression is 0–9, for anxiety is 0–7 and for stress is 0–14. Scores above these ranges suggest an increasing severity of symptoms. The questionnaires were validated and adapted for the Greek language and population.^19^

Data analysis included descriptive statistics to summarise demographic and psychological variables. Group comparisons used the chi-square test for categorical variables, such as marital status, and the Mann–Whitney *U*-test for YSQ and DASS-21 scores. Analysis of variance with Bonferroni adjustment was applied for DASS-21 subscale comparisons. A mediation analysis examined the indirect effects of societal pressures on psychological distress via EMS, employing maximum-likelihood estimation and delta method standard errors with JASP software (version 0.16.3 for Windows; JASP Software, University of Amsterdam, The Netherlands; https://jasp-stats.org/download/; *α* = 0.05).

## Results

### Demographic characteristics

The study examined the demographic characteristics of 1341 Greek women aged 30–50 years, of whom 503 were without children and 838 with child/children (Table 1).

**Table 1:** Demographic and sociodemographic comparisons between child-free women and women with children

Variable	Category	Child-free women (*n* = 503)		Mothers (*n* = 838)		*χ*²/*P*-value
		*n*	%	*n*	%	
Age (years)						*χ*² = 120.339, *P* < 0.001
	30–35	262	52.1	197	23.5	
	36–40	138	27.4	353	42.0	
	41–45	71	14.1	235	28.0	
	46–50	32	6.4	55	6.5	
Education						*χ*² = 10.048, *P* = 0.018
	Secondary	31	6.2	72	8.6	
	Tertiary	192	38.2	363	43.2	
	Postgraduate/doctoral	277	55.1	394	46.9	
Occupation						
	Public sector	126	25.0	203	24.2	
	Private sector	205	40.8	321	38.2	
	Self-employed/freelancer	124	24.7	188	22.4	
	Home-maker	4	0.8	50	6.0	
	Unemployed	44	8.7	78	9.3	
Household income^a^						*χ*² = 182.072, *P* < 0.001
	<€1000	131	26.0	98	11.7	
	€1000–1500	145	28.8	219	26.1	
	≥€1500	108	21.5	460	54.8	
Relationship status						
	Single	188	37.4	–		
	In relationship/married	302	60.0	444	52.9	
	Separated/divorced/widowed	13	2.6	–	–	
Sexual orientation						*χ*² = 28.219, *P* < 0.001
	Heterosexual	465	92.4	824	98.1	
	LGBTQ+	38	7.6	16	1.9	

LGBTQ+, lesbian, gay, bisexual, transgender and queer/questioning.

a. Income represents individual income for non-mothers and family income for mothers, due to different household compositions.

**Table 2:** Depression, Anxiety and Stress Scale 21 (DASS-21) mental health outcome scores^a^

Scale	Category	Child-free women (*n* = 503)	Mothers (*n* = 838)
Depression			
	Mean (s.d.)	12.95 (11.32)	9.90 (10.32)
	Normal (0–9)	239 (47.5%)	500 (59.6%)
	Mild (10–13)	92 (18.3%)	121 (14.4%)
	Moderate (14–20)	92 (18.3%)	121 (14.4%)
	Severe (21–27)	45 (8.9%)	62 (7.4%)
	Extremely severe (28+)	35 (7.0%)	34 (4.1%)
Anxiety			
	Mean (s.d.)	9.91 (10.28)	7.47 (8.92)
	Normal (0–7)	270 (53.7%)	539 (64.3%)
	Mild (8–9)	44 (8.7%)	78 (9.3%)
	Moderate (10–14)	85 (16.9%)	113 (13.5%)
	Severe (15–19)	42 (8.3%)	58 (6.9%)
	Extremely severe (20+)	62 (12.3%)	50 (6.0%)
Stress			
	Mean (s.d.)	16.04 (11.18)	16.61 (11.12)
	Normal (0–14)	254 (50.5%)	418 (49.9%)
	Mild (15–18)	73 (14.5%)	139 (16.6%)
	Moderate (19–25)	103 (20.5%)	164 (19.6%)
	Severe (26–33)	45 (8.9%)	78 (9.3%)
	Extremely severe (34+)	28 (5.6%)	39 (4.7%)

a. DASS-21 cut-off values: depression (normal, 0–9; mild, 10–13; moderate, 14–20; severe, 21–27; extremely severe, 28+); anxiety (normal, 0–7; mild, 8–9; moderate, 10–14; severe, 15–19; extremely severe, 20+); stress (normal, 0–14; mild, 15–18; moderate, 19–25; severe, 26–33; extremely severe, 34+).^18^

**Table 3:** Summary of attitudes, concerns and mental health outcomes in child-free women

Category/variable	Specifics	Statistics/findings	Mental health outcomes	Schema mediation/significant paths
Attitudes about motherhood^a^	Want child(ren)	39.6%		
	Uncertain about motherhood	38.2%		
	Do not want child(ren) (child-free by choice)	12.1%		
	Prefer parenthood within relationship	63.6%		
	Concerns intensified beyond age 30 years	44.9%	Higher stress (mean 19.45, s.d. 11.69)	Schemas amplify stress when major concerns persist beyond age 30 years
	Thinks about motherhood ‘as before’ or ‘not concerned’	Subgroup	Lower stress (mean approximately 13.9)	
Motherhood and childlessness concerns^b^	Partner suitability ranked very/extremely important	67.2%		Emotional deprivation mediates increased depression, anxiety and stress in ‘finding suitable partner’
	‘Biological clock’ anxiety very/extreme	51.7%		
	Extremely concerned about life changes	35.6%		
	Extremely concerned about responsibility	37.6%		
	Fear of regret if remaining childless	49.8%		Mistrust/abuse, vulnerability mediates increased stress, anxiety and depression in ‘regret’
	Concerns beyond age 30 years: ‘pressure a lot/affect decisions’	Subgroup	Higher stress (mean 19.45, s.d. 11.69)	
Mental health outcomes^c^	Mean depression (DASS-21)	12.95 (s.d. 11.32)	47.5% in normal range	Depressive symptoms exacerbated by schemas emotional deprivation, mistrust/abuse
	Mean anxiety (DASS-21)	9.91 (s.d. 10.28)	53.7% in normal range	Anxiety symptoms stronger with schemas vulnerability, emotional deprivation
	Mean stress (DASS-21)	16.04 (s.d. 11.17)	50.5% in normal range	Highest stress in those reporting motherhood pressures beyond age 30 years
	Severe/extreme depression	7%		
	Severe/extreme anxiety	12.3%		
	Severe/extreme stress	5.6%		
Statistical/schema associations^d^	Higher concerns/pressure beyond age 30 years – stress	Significant (*P* < 0.05)	Increased stress	High concern + high schema scores (deprivation, mistrust): amplify negative outcomes
	Depression by concern group	Not significant (*P* = 0.328)		
	Anxiety by concern group	Not significant (*P* = 0.262)		
	Mediation effects (significant *β*, *P* < 0.05)	*β* = 0.113–0.181	Increased depression, anxiety and stress	Emotional deprivation (finding partner, motherhood); mistrust/abuse (regret, childlessness)
	Schemas–concern interaction	Combined, further amplifies outcomes	Increased risk for depression, anxiety, stress	Multiple pathways: emotional deprivation + mistrust/abuse results in worse stress, depression, anxiety

DASS-21, Depression, Anxiety and Stress Scale 21.

a. Mediation analysis shows that the schemas emotional deprivation and mistrust/abuse significantly increase depression, anxiety and stress regarding concerns about motherhood, partner suitability and fear of regret.

b. Women beyond 30 years of age reporting intensified motherhood concerns and societal pressure have statistically higher stress scores, mediated by schemas.

c. Depression and anxiety are also amplified when high levels of concern co-occur with strong maladaptive schemas, although only stress showed a significant association by concern subgroup.

d. High concern–schema interaction represents the most vulnerable group regarding psychological distress.

**Table 4:** Young Schema Questionnaire (YSQ) comparison of early maladaptive schemas between child-free women and mothers

Schema	Non-mothers (*n* = 503)				Mothers (*n* = 838)				*P*-value
	Mean	s.d.	Median	Range	Mean	s.d.	Median	Range	
Emotional deprivation	2.19	1.12	1.80	5.00	2.13	1.11	1.80	5.00	0.329
Abandonment	2.49	1.17	2.40	5.00	2.16	1.04	2.00	5.00	**<0.001**
Mistrust/abuse	2.25	1.14	2.00	5.00	2.10	1.09	1.80	5.00	**0.013**
Social isolation	2.32	1.20	2.00	5.00	2.25	1.21	1.80	5.00	0.164
Defectiveness/shame	1.72	0.91	1.40	4.40	1.59	0.81	1.20	4.60	**0.008**
Failure to achieve	1.81	0.99	1.40	4.80	1.80	1.01	1.40	4.80	0.451
Dependence	2.39	0.69	2.20	3.80	2.36	0.69	2.20	4.20	0.289
Vulnerability	2.11	0.99	1.80	4.80	2.18	1.04	2.00	4.60	0.367
Enmeshment	2.13	0.99	2.00	5.00	1.97	0.99	1.60	5.00	**<0.001**
Subjugation	2.25	1.09	2.00	4.60	2.08	1.02	1.80	4.60	**0.006**
Self-sacrifice	3.36	1.11	3.20	5.00	3.47	1.10	3.40	5.00	0.059
Emotional inhibition	2.43	1.08	2.20	4.40	2.26	1.12	2.00	4.80	**0.001**
Unrelenting standards	3.52	1.11	3.60	5.00	3.45	1.11	3.40	5.00	0.230
Entitlement	2.83	0.94	2.80	5.00	2.84	0.94	2.80	4.80	0.958
Insufficient self-control	2.76	0.97	2.80	4.60	2.68	1.02	2.60	5.00	0.090
Approval-seeking	3.05	1.14	3.00	5.00	2.88	1.16	2.80	5.00	**0.005**
Negativity/pessimism	2.72	1.18	2.60	5.00	2.59	1.20	2.40	4.80	**0.034**
Punitiveness	2.62	1.01	2.60	5.00	2.46	0.93	2.40	4.60	**0.006**

YSQ scores range from 1 to 6, with higher scores indicating stronger schema endorsement.

Scores ≥4.0 typically indicate clinically significant schema activation.

Analysis based on Mann–Whitney *U*-tests due to non-normal distributions.

Bolded *P*-values indicate statistical significance (*P* < 0.05).

Among child-free women, most were aged 30–35 years with a small percentage (6.4%) aged 46–50 years. Over half (55.1%) had a postgraduate or doctoral degree. About 41% worked in the private sector. Ethnicity was not recorded because the sample was ethnically homogeneous. Regarding income, roughly 29% earned between €1000 and 1500 per month while 26% earned less than €1000. Most identified as heterosexual (92.6%). Regarding participants’ gynaecological history, nearly 92% had no history of abortion and about 90.5% had had no miscarriages. A small percentage (5.4%) had frozen eggs and over half (57.5%) did not plan to. Regarding COVID-19, about 80% were fully vaccinated; only 1.2% cited fertility concerns as a reason for not becoming vaccinated. Regarding desire for motherhood, approximately 40% of child-free women wanted children, 38% were unsure and only 12% explicitly did not want them. Consequently, the sample predominantly represents women who are child-free by circumstance rather than by choice. In regard to their relationship status, 60% stated that they were in a relationship/marriage, 37.4% were single and 2.6% were separated/divorced/widowed.

Most mothers in the study were aged between 36 and 40 years, making up 42% of the group, and about 24% were aged 30–35 years. Nearly half (47%) had a university or doctoral degree and over half (55%) earned at least €1500 per month. Most (98%) identified as heterosexual. Among these mothers, 82% had never had an abortion and 89% had conceived naturally. A small percentage (3%) had used frozen eggs. Regarding COVID-19 vaccination, 72% were fully vaccinated while 2% had avoided vaccination due to fertility concerns. Most women had one child (53%), some had two (41%) and several had three or more (6%). They mostly had their children naturally (89%), with others using in vitro fertilisation (8%) or adoption/foster care (0.1%). When asked whether they wanted more children, 41% said no and 25% said yes but wouldn’t, mainly due to financial or practical reasons.

Statistical analyses indicated that mothers were older (*χ* ^2^ = 120.339, *P* < 0.001) and had a higher household income (*χ*² = 182.072, *P* < 0.001). Child-free women were more likely to identify with diverse sexual orientations (*χ*² = 28.219, *P* < 0.001).

### Mental health outcomes

Women without children had higher average scores for depression (mean 12.95, s.d. = 11.32) and anxiety (mean 9.91, s.d. = 10.28) compared with mothers, who scored an average of 9.89 (s.d. = 10.32) for depression and 7.47 (s.d. = 8.92) for anxiety. Stress levels showed no significant difference between groups, with mean scores of 16.04 (s.d. = 11.17) for child-free women and 16.60 (s.d. = 11.12) for mothers.

While most participants scored within the normal range on the DASS-21 subscales, a notable subset showed clinically significant symptoms. Among child-free women, 7.0% had extremely severe depression scores, 12.3% exhibited extremely severe anxiety symptoms and 5.6% reported extremely severe stress. In comparison, mothers had lower scores in the extremely severe range category for depression (4.1%), anxiety (6.0%) and stress (4.7%). These findings indicate greater psychological distress in child-free women compared with mothers (Table 2).

Subgroup analyses showed significant links between occupational status and mental health outcomes. Child-free women in the private sector reported higher depression levels (14.94 (s.d. = 12.25)) compared with public employees (11.14 (s.d. = 10.56)) and self-employed or freelancers (10.84 (s.d. = 9.92)). However, child-free women who were home-makers scored higher for anxiety (17.50 (s.d. = 5.26)) and stress (18.50 (s.d. = 8.22)).

Respectively, home-maker and unemployed mothers had higher scores for depression (14.88 (s.d. = 11.74)); 12.56 (s.d. = 10.72)) and stress (19.68 (s.d. = 11.36)); 16.95 (s.d. = 11.29)) than public employees (depression 8.35 (s.d. = 9.62)); stress 14.56 (s.d. = 11.17)).

Regarding income, mothers earning €1500 or more per month reported lower depression (8.58 (s.d. = 9.61)), anxiety (6.46 (s.d. = 8.07)) and stress (15.63 (s.d. = 11.13)) compared with those earning €1000–1500 and those with a basic salary (i.e. <€1000).

### Attitudes towards motherhood

Table 3 presents attitudes related to motherhood. Among women without children, 39.56% desired children, 38.17% were uncertain and 12.13% did not want children. A majority (63.62%) preferred having children within a relationship or marriage, while 21.27% considered sperm donors and 15.11% contemplated single parenthood. Beyond age 30 years, 44.93% of women without children reported intensified thoughts about motherhood. Key concerns included finding a suitable partner (67.2% rated this as ‘very/extremely’ important), biological clock anxiety (51.69% were ‘very/extremely’ concerned), life changes (35.59% were ‘extremely’ concerned) and responsibility (37.57% were ‘extremely’ concerned).

Among mothers, the number of children varied: 52.9% had one child, 40.8% two children and 6.3% three or more children; 40.7% did not want more children, mainly due to financial or logistical barriers (25.1%). After becoming mothers, 78.6% considered a supportive partner critical while 34.4% saw motherhood as a personal achievement. Notably, 80.7% expressed no regret about having children.

Partner suitability, responsibility, fulfilment and regret were significantly evaluated both before and after becoming a mother. Notably, 95% of mothers considered partner suitability as ‘very/extremely’ important, compared with 67.2% of women before motherhood. Mothers rated responsibility concerns as ‘very/extremely’ important, at 69.1%, versus 58.3% of women without children. Furthermore, 91% of mothers found child-bearing fulfilling, compared with 65.6% of women without children. Additionally, only 8.9% of mothers expressed regret after motherhood, while 49.8% of women without children feared regret if they remained childless.

Two results stood out when analysing variations in the desire for motherhood based on the three DASS dimensions. Women trying to conceive reported statistically significantly lower depression scores (9.65 (s.d. = 8.11)) than those uncertain about motherhood (14.72 (s.d. = 11.98)), those wanting children (12.22 (s.d. = 10.83)) and those not wanting children (12.52 (s.d. = 12.33)).

The analysis also revealed significant differences among women regarding motherhood beyond the age of 30 years. Those women without children who felt that motherhood ‘pressures them a lot and affects their life decisions’ had higher stress (19.45 (s.d. = 11.69)) than those ‘thinking about it as often as before’ (13.87 (s.d. = 10.20)) and those ‘not thinking about it’ (13.98 (s.d. = 10.61)). The terminology reported above represents direct quotations from the custom questionnaire evaluating motherhood concerns. In contrast, depression (*P* = 0.328) and anxiety (*P* = 0.262) showed no significant difference.

### YSQ

The study compared psychological traits between child-free women and mothers using YSQ, analysed using the Mann–Whitney *U*-test (*α* = 0.05). Significant differences (*P* < 0.05) were identified, with child-free women showing higher scores in several areas: abandonment (2.40 *v.* 2.00), mistrust/abuse (2.00 *v.* 1.80), defectiveness/shame (1.40 *v.* 1.20), undeveloped self/enmeshment (2.00 *v.* 1.60), subjugation (2.00 *v.* 1.80) and emotional inhibition (2.20 *v.* 2.00). They also scored higher in approval-seeking (3.00 *v.* 2.80), negativity/pessimism (2.60 *v.* 2.40) and punitiveness (2.60 *v.* 2.40) (Table 4).

### Mediating effects of EMS in child-free women

The mediation analysis examined how deeply ingrained thought patterns (EMS) mediate the relationship between concerns about motherhood or childlessness and mental health outcomes such as depression, anxiety and stress.

Among these schemas, emotional deprivation and mistrust/abuse stood out as the most influential. Emotional deprivation worsened mental health across both areas: for motherhood concerns, worries about finding a suitable partner were linked to increased depression (*β* = 0.111), anxiety (*β* = 0.091) and stress (*β* = 0.096), while distress related to childlessness – such as feelings of underachievement (*β* = 0.149 for depression) or regret (*β* = 0.161 for depression) – was intensified by this schema. Mistrust/abuse similarly contributed to negative outcomes, especially in childlessness concerns, where fears of failing others’ expectations led to higher depression (*β* = 0.113), and regret associated with mistrust raised stress to the highest level observed (*β* = 0.185).

Entitlement plays a dual role. Lower scores on these traits were linked to increased depression related to motherhood concerns, such as permanent life changes (*β* = 0.147) and difficulties in balancing career and motherhood when childless (*β* = 0.129). On the other hand, stronger entitlement beliefs served as a protective factor: women without children who had higher entitlement scores experienced less depression (*β* = −0.065), anxiety (*β* = −0.085) and stress (*β* = −0.091) when worried about time passing without children. Vulnerability, however, consistently predicted higher anxiety in women without children, especially fears of missing out (*β* = 0.141) and regret (*β* = 0.163). When vulnerability was combined with mistrust or perfectionism (unrelenting standards), it also increased stress, particularly in relation to regret (*β* = 0.185).

Schemas related to self-regulation and perfectionism further increased stress. Insufficient self-control/discipline heightened stress in motherhood concerns (e.g. *β* = 0.138 for permanent changes) and issues related to childlessness. (e.g. *β* = 0.141 for failed expectations), while unrelenting standards amplified stress in women without children fixated on unmet goals, such as regret (*β* = 0.185) or fears of missing out (*β* = 0.167). Less dominant schemas also played notable roles: emotional inhibition, related to motherhood concerns about permanent changes, was linked to elevated depression (*β* = 0.147); and dependence, when paired with mistrust, modestly increased stress (*β* = 0.138) (Supplementary material).

## Discussion

This study explored differences among 1341 Greek women without children and mothers aged 30–50 years, highlighting demographic disparities, mental health outcomes and the impact of EMS. The findings show how schemas can shape women’s responses to reproductive expectations, suggesting new strategies to empower them to make independent choices in pronatalist societies.

Mothers tended to be older and financially stable, reflecting the trend of delayed child-bearing among financially secure women.^20^ In contrast, child-free women, despite higher education, faced greater economic constraints and societal stigma related to the biological clock. While stress levels were similar, stressors varied. Child-free women deal with existential uncertainties and societal pressures, while mothers face challenges managing caregiving demands and work–life balance. Occupational settings had shaped mental health, with child-free women in high-pressure jobs experiencing more depression and anxiety while home-maker mothers suffered poorer mental health.

Financial stability emerged as a key buffer for maternal well-being, supporting research that shows its role in reducing parenting stress, because women with higher income had lower scores for depression, anxiety and stress.

It is important to note that, while the child-free women in this study showed higher levels of depression and anxiety, causality cannot be confidently established from cross-sectional data. This link may have resulted from existing mental health issues that had affected reproductive choices or life paths. Alternatively, depressive symptoms might be connected to current challenges often faced by women at this stage of life, including strong societal pressures related to reproduction, struggles with forming or maintaining close relationships, financial difficulties or workplace stress.^21,22^ The interaction of these factors probably added to the psychological distress observed. Therefore, rather than assuming a direct cause-and-effect relationship, it is important to see this as a complex phenomenon shaped by a web of biological, psychological and sociocultural factors.

Child-free women’s concerns about motherhood – partner-searching, biological clock anxiety and fears of life changes – reflect pronatalist norms linking womanhood to motherhood.^23^ Many child-free women value relational stability when considering parenthood, possibly due to societal preferences for child-bearing within partnerships. The study shows that women over 30 years of age feel greater stress about motherhood, especially when societal pressures appear intrusive, and in particular for those who are child-free unintentionally. This supports findings that age-related pronatalist expectations increase anxieties about the biological clock, raising stress even without clinical depression or anxiety. In contrast, mothers cited economic barriers among the reasons for limiting family size, echoing global trends where financial instability restricts fertility aspirations.^24^ They also reported higher fulfilment and lower regret, reflecting cultural ideals that elevate motherhood as a core achievement for women.^1^

The study identifies EMS as mediators of mental health outcomes. Key findings emphasise the significance of the schemas emotional deprivation and mistrust/abuse. Emotional deprivation has been shown to negatively impact mental health, especially among women grappling with concerns about motherhood and childlessness. This supports earlier studies that link unmet emotional needs to higher rates of depression and anxiety.^25^ For example, the worry of finding a compatible partner is linked to higher emotional distress (e.g. *β* = 0.111 for depression). This suggests that societal pressures for a ‘traditional’ family structure can intensify, especially among child-free women with emotional deprivation schemas. Furthermore, distress related to being childless, often felt as underachievement or regret (*β* = 0.149–0.161), illustrates how societal beliefs that equate fulfilment with parenthood can result in harsh self-criticism, particularly among those who experience emotional deprivation. If the survey had been carried out solely on women who were child-free by choice, the results would probably have been different. The mistrust/abuse schema increases negative outcomes, especially when women feel that they have not met societal expectations (e.g. *β* = 0.113 for depression). This supports theories suggesting that a mistrustful mindset boosts feelings of social rejection, raising stress levels for those who pursue non-traditional life choices.^26^

The link between being child-free and feeling mentally stressed is similarly complicated: the study showed that women without children often have higher anxiety levels. However, we cannot definitively establish that the absence of children is the cause of that anxiety. Only about 12.13% of these women said that they didn’t want children by choice, and therefore most probably many of them do want children but face obstacles in having them. This suggests that their stress may not be solely due to their non-traditional life choice. Other factors, including difficult childhood, relationship issues and social challenges, might influence both their decision to stay child-free and their mental health. Past experiences and societal expectations can impact both their reproductive choices and feelings of stress.

A key finding of the study is the dual role of entitlement. Lower entitlement scores were linked to increased depression related to motherhood concerns (for example, *β* = 0.147 for permanent life changes), indicating a decline in self-efficacy during identity transitions. In contrast, stronger beliefs in entitlement helped lessen distress among women who worried about not having children as time went on (with *β* ranging from −0.065 to −0.091). This protective effect may have arisen from resilience against internalised stigma, suggesting that a sense of deservingness can reduce negative societal views surrounding child-free lifestyles. This complexity demonstrates that certain maladaptive traits, such as entitlement, can sometimes have adaptive benefits, a phenomenon that requires further investigation.

Understanding the dual role of entitlement can inform psychological interventions. If entitlement helps women resist internalised stigma or self-criticism about motherhood, therapy can strengthen these resilience factors. Research should explore how entitlement interacts with other mental processes to help clinicians balance its protective effects with potential drawbacks, improving mental health outcomes.

Vulnerability reliably predicts anxiety related to childlessness concerns, particularly regarding fears of missing out (*β* = 0.141) or regret (*β* = 0.163). These findings support cognitive models that highlight catastrophising as a key mechanism in anxiety disorders. Importantly, the interaction between vulnerability and unrelenting standards (e.g. *β* = 0.185 for stress) indicates that perfectionistic tendencies worsen distress by framing childlessness as a personal failure, intensifying self-imposed pressure. Additionally, schemas related to self-regulation (e.g. insufficient self-control) and perfectionism increased stress across various concerns, highlighting the importance of addressing rigid self-expectations in therapy.

Nevertheless, distinguishing schema-driven perceptual distortions from actual social stressors or other sources of distress is challenging. EMS can interact with genuine societal demands, making it difficult to parse whether distress stems primarily from external pressures or the filtering effect of maladaptive beliefs. Additionally, comorbid life stressors such as relationship difficulties, financial insecurity or health problems often coexist and compound psychological distress, further complicating attribution.

### Limitations

Our findings primarily reflect the experiences of women who were mostly involuntarily child-free at the given time point; if the group had consisted entirely of women who were intentionally child-free by choice, the psychological profiles might have been different.

The study’s cross-sectional design limits causal inferences, and its homogeneous urban sample restricts generalisability. Rural populations and lower-income women’s groups may experience pronatalist pressures differently, because financial precarity could heighten anxieties concerning the biological clock.^24^ The high proportion of participants with a postgraduate degree may also have limited generalisability, because educational attainment can influence reproductive choices and mental health. Sexual minority participants were underrepresented. Research consistently demonstrates that sexual minority women experience higher rates of mental health issues such as depression, anxiety and suicidality compared with heterosexual women.^27,28^ These disparities are often explained through minority stress theory, which states that sexual minorities face ongoing stressors – including discrimination, stigma and anticipation of rejection – that negatively impact their psychological well-being.^29^ In regard to reproductive health, sexual minority women may encounter additional challenges such as reproductive coercion, limited access to quality healthcare and societal heteronormative pressures that make decisions about motherhood and child-bearing more difficult.^30,31^ These overlapping challenges highlight the need to consider sexual minority status separately in reproductive and mental health research so that tailored, culturally competent interventions can be developed to meet their specific needs.

### Conclusions

This study provides new insights into the psychological links between societal pressures and mental health. Recognising EMS as mediators shifts the focus from external pronatalist influences to interventions aimed at internal cognitive frameworks. Cognitive–behavioural therapies that target schemas such as emotional deprivation or self-sacrifice could reduce distress, especially for ambivalent women without children. Clinically, these insights support schema-based interventions to help women make informed decisions about parenthood, ultimately promoting mental well-being across different life paths.

Future, long-term studies in diverse groups of people, and a combination of qualitative and quantitative methods, will provide a better understanding. Qualitative research can explain how cultural factors affect the development of schemas. Additionally, understanding how EMS fit into different cultural contexts can help support women’s reproductive rights and ensure fair mental healthcare for all.

In conclusion, this study highlights the importance of interventions that target maladaptive schemas to reduce the emotional burden of reproductive concerns. By addressing societal narratives and cognitive patterns, policy-makers and clinicians can better support women navigating the complex relationships among choice, identity and well-being.

## Supplementary material

The supplementary material for this article is available online at https://doi.org/10.1192/bji.2025.10093

## Author contributions

M.E.: conceptualisation, study design, participant recruitment, data collection, writing – original draft. S.I.B.: methodology, statistical analysis, data visualisation (tables/figures). I.I.-B.: project administration, ethical oversight, interpretation of results. M.S.: supervision, validation, review, editing.

## Funding

This research received no specific grant from any funding agency in the public, commercial or not-for-profit sectors.

## Declaration of interest

M.S. is a member of the *BJPsych International* editorial board but was not involved in any stage of the peer review or editorial decision-making for this article. The other authors have no further interests to declare.

## Ethical standards

This study was approved by the Bioethics Committee of the Medical Department of Aristotle University of Thessaloniki, Greece (approval no. 2022-23/0379).

The authors assert that all procedures contributing to this work comply with the ethical standards of the relevant national and institutional committees on human experimentation, and with the Helsinki Declaration of 1975 as revised in 2013.

## Consent to participate

Prior to their involvement in the study, written informed consent was obtained from all participants.

## Consent to publish

Participants provided consent for the publication of anonymised data in this manuscript.

## Supporting information

Evangelopoulou et al. supplementary material 1 Evangelopoulou et al. supplementary material

Evangelopoulou et al. supplementary material 2 Evangelopoulou et al. supplementary material
