# Supplementary material for: Early maladaptive schemas as mediators of pronatalist pressures: comparative study of psychological distress in child-free women and mothers
Source: BJPsych Int. 2026 Feb 9;23(2):79–89. doi: 10.1192/bji.2025.10093 (PMC13732258; doi:10.1192/bji.2025.10093)
Supplement: Supplementary file 1 [file S2056474025100937sup001.docx]

Custom Questionnaire addressed to Child-free Women and Mothers

Custom Questionnaire addressed to childfree women and mothers assessing pre- and post-motherhood concerns.

| **Child-free Women** | **Mothers** |
| --- | --- |
| When it comes to having a child, my biggest concern is finding a suitable partner. | After having a child or children, I believe having a suitable partner was important. |
| When it comes to having a child, my biggest concern is the passage of time. | After having a child or children, what concerns me most is the passage of time. |
| When it comes to having a child, what concerns me most are the permanent changes it will bring. | After having a child or children, what concerns me the most are the permanent changes they have brought. |
| When it comes to having a child, my biggest concern is the responsibility. | After having a child or children, the responsibility concerns me the most. |
| What concerns me most about **not** having a child is the feeling that I will not have achieved something important. | After having a child or children, I feel that I have achieved something important. |
| What concerns me most about **not** having a child is the thought that I have prioritized other areas, such as my career and personal development. | After having a child or children, I think I have not been able to prioritize other areas as much, such as my career and personal development. |
| What concerns me most about not having a child is the thought that I failed others' expectations. | After having a child or children, I feel that I have fulfilled societal expectations regarding motherhood. |
| What concerns me the most about **not** having a child is the thought that something will be missing. | After having a child or children, I think that something has been completed. |
| What concerns me the most about **not** having a child is the thought that I will regret it. | After having a child or children, I do not regret it. |

Note: They used for each question a 5-point Likert scale:

1. "Not at all"
2. "Slightly"
3. "Moderately"
4. "Very"
5. "Extremely"
