# Supplementary material for: Early maladaptive schemas as mediators of pronatalist pressures: comparative study of psychological distress in child-free women and mothers
Source: BJPsych Int. 2026 Feb 9;23(2):79–89. doi: 10.1192/bji.2025.10093 (PMC13732258; doi:10.1192/bji.2025.10093)
Supplement: Supplementary file 2 [file S2056474025100937sup002.docx]

**Table 5: Mediation Results**

**Significant Mediation Effects of Early Maladaptive Schemas**

**A. DEPRESSION OUTCOMES**

| **Schema** | **Concern Type** | **Specific Concern** | **β** | **p-value** |
| --- | --- | --- | --- | --- |
| **Emotional Deprivation** | Motherhood | Finding a partner | 0.113 | <0.001 |
| **Emotional Deprivation** | Childlessness | Not accomplishment | 0.142 | 0.001 |
| **Mistrust/Abuse** | Childlessness | Failed expectations | 0.122 | 0.001 |

**B. ANXIETY OUTCOMES**

| **Schema** | **Concern Type** | **Specific Concern** | **β** | **p-value** |
| --- | --- | --- | --- | --- |
| **Emotional Deprivation** | Motherhood | Finding a partner | 0.113 | <0.001 |
| **Vulnerability** | Childlessness | Something missing | 0.141 | 0.001 |

**C. STRESS OUTCOMES**

| **Schema** | **Concern Type** | **Specific Concern** | **β** | **p-value** |
| --- | --- | --- | --- | --- |
| **Emotional Deprivation** | Motherhood | Finding a partner | 0.141 | <0.001 |
| **Mistrust/Abuse** | Childlessness | Regret | 0.167 | 0.001 |
| **Insufficient Self-Control** | Motherhood | Permanent changes | 0.181 | <0.001 |

**Notes:**

- Only significant mediation effects (p<0.05) are reported
- β represents standardized mediation effect size
- Analysis based on bootstrap methods with 95% confidence intervals
